# Cyclic Mechanical Stretch Ameliorates the Degeneration of Nucleus Pulposus Cells through Promoting the ITGA2/PI3K/AKT Signaling Pathway

**DOI:** 10.1155/2021/6699326

**Published:** 2021-03-16

**Authors:** Dandan Wang, Yuanzhen Chen, Shengnan Cao, Pengcheng Ren, Haojun Shi, Huazhong Li, Liangyu Xie, Weimin Huang, Bin Shi, Jinxiang Han

**Affiliations:** ^1^Bone Biomechanics Engineering Laboratory of Shandong Province, Neck-Shoulder and Lumbocrural Pain Hospital of Shandong First Medical University, Shandong Medicinal Biotechnology Center, Shandong First Medical University & Shandong Academy of Medical Sciences, Jinan 250062, China; ^2^Shandong University of Traditional Chinese Medicine, Jinan 250014, China; ^3^Second Clinical Medical College, Henan University of Chinese Medicine, Henan, China; ^4^960th Hospital of PLA, Jinan 250031, China

## Abstract

**Background:**

Intervertebral disc degeneration (IVDD) is one of the major causes of low back pain and motor deficiency. Nucleus pulposus (NP) degeneration plays a key role in the process of IVDD. The mechanical and biological interactions involved in NP degeneration have not been elucidated. The present study is aimed at investigating the effect and mechanism of cyclic mechanical stretch in regulating the function and degeneration of NP cells.

**Methods:**

NP cells were subjected to cyclic tensile stress (10% deformation) of 0.1 Hz for 8640 cycles. Cell proliferation was conducted through the MTT assay. The cell cycle and apoptosis were detected by flow cytometry. A gene expression profile chip was used to analyze the differentially expressed genes between the tensile stress group and the control group. Enrichment analysis of Gene Ontology (GO) annotation and signaling pathways were analyzed. Western blot and RNA interference were carried out to investigate the role of the ITGA2/PI3K/AKT pathway in the effect of cyclic mechanical stretch on NP cells.

**Results:**

NP cells exhibited a greater (*P* < 0.05) growth rate in the tensile stress group compared to the control group. Cyclic mechanical stress significantly promoted the cell cycle transition of NP cells from the S phase to the G2/M phase. A fewer proportion of apoptotic cells were found in the tensile stress group (*P* < 0.05), indicating that cyclic mechanical stretch inhibits NP cell apoptosis. Microarray analysis revealed 689 significant differentially expressed genes between the two groups (*P* < 0.05), of which 333 genes were upregulated and another 356 genes were downregulated. Cyclic mechanical stretch altered the expression of 31 genes involved in the ITGA2/PI3K/AKT pathway and remarkably promoted this pathway in NP cells. Downregulation of ITGA2 and AKT further demonstrated that the PI3K/AKT pathway was responsible for the proliferation and COL2A1 expression of NP cells upon cyclic mechanical stretch.

**Conclusions:**

Cyclic mechanical stretch promoted the proliferation and cell cycle and reversely inhibited the apoptosis of NP cells. Cyclic mechanical stretch promoted COL2A1 expression and ameliorated the degeneration of NP cells via regulation of the ITGA2/PI3K/AKT signaling pathway. Our results may provide a potential target and a possibility of IVDD disease treatment by ameliorating the degenerative changes.

## 1. Introduction

Intervertebral disc degeneration (IVDD) is frequently associated with low back pain (LBP), which is one of the most prevalent musculoskeletal problems and the leading cause of disability [1, 2]. IVDD causes instability, stenosis, and deformity of the spine and is considered an aberrant, pathological, and cell-mediated response leading to progressive structural failure and pain [3]. Despite the global prevalence and high economic burden associated with IVDD diseases [4], the pathogenesis and molecular mechanisms leading to IVDD have not been fully elucidated.

The mature healthy intervertebral disc (IVD), a connective tissue located between the vertebrate bodies of the vertebrae, is composed of three components: nucleus pulposus (NP), annulus fibrosus (AF), and cartilaginous endplates (CEPs) that anchor onto the vertebrae [5]. The main functions of IVD include transmission and absorption of mechanical loadings onto the vertebral column in order to maintain the motional function that allows extension, flexion, lateral bending, and axial rotation [6, 7]. The NP is a highly hydrated gelatinous mass that comprises the chondrocytes and proteoglycans incorporating an irregular network of elastin fibers and type II collagen [8]. Overall, the healthy NP generates intradiscal pressure that separates the vertebrae above and below, maintains the tension of the AF, and distributes the pressure evenly across two adjacent CEPs [8]. Degeneration of NP has been considered a vital step in IVDD. A degenerated NP becomes an unorganized mass of fibrous tissue with altered extracellular matrix (ECM) components that has mainly lost its capacity of binding water [9]. The pressure within the degenerated NP dwindles, and the disc height decreases, resulting in IVDD and spinal biomechanical instability [10]. The NP undergoes the highest degree of remodeling during IVDD.

A wealth of complex factors give rise to disc degeneration and commonly involve synergistic interactions between biomechanics and biological mechanisms [8]. During their interactions, mechanical factors, cell biological metabolism response, and the water-binding ECM play important roles in degeneration. Some changes occur in degenerating NP cells; for example, the predominant cell type changes from notochordal cells to chondrocyte-like cells, the ECM changes from anabolism to catabolism, and inflammatory mediators increased [11–14]. The phosphatidylinositol 3-kinase (PI3K)/AKT pathway governs many fundamental cellular functions and participates in regulating transcription, translation, cell proliferation, differentiation, cell cycle, and apoptosis [15, 16]. The PI3K/AKT pathway has been reported to participate in regulating NP cell proliferation, apoptosis, senescence, and ECM metabolism, and it has also been shown to be significantly associated with NP degeneration [17–19]. However, the mechanics and biological interaction mechanism in regulating the degeneration of NP have not yet been explored in depth.

In this research, we investigated the effect of cyclic mechanical stretch on the biological function of NP cells and the role of the ITGA2/PI3K/AKT pathway in response to the effect of cyclic mechanical stretch on NP cells. The results from our study may provide potential targets and the possibility of reversing the degenerative changes in IVDD.

## 2. Materials and Methods

### 2.1. Ethics Statement

This study was approved by the Ethics Committee of Shandong First Medical University & Shandong Academy of Medical Sciences. Written informed consent was obtained from every study participant.

### 2.2. Human NP Tissues

Human NP tissues were obtained from patients (17 donors, 30-60 years, and Pfirrmann degeneration grade 3–4 [20]) who underwent percutaneous endoscopic discectomy in the Neck-Shoulder and Lumbocrural Pain Hospital of Shandong First Medical University and the 960^th^ Hospital of PLA, China. Patients with spinal tumors, spinal tuberculosis, or spondylodiscitis were excluded from participation in this study.

### 2.3. Isolation and Culture of NP Cells

NP tissues were washed thrice with phosphate-buffered saline (PBS) solution and cut into 1 to 2 mm^3^ pieces under sterile conditions. The tissues were digested with 0.25% trypsin (Gibco, USA) for 30 min in a water bath at 37°C. The tissue samples were centrifuged at 1200 rpm for 5 min. The obtained precipitates were then digested by incubation with 0.1% type II collagenase for 3 h in a water bath at 37°C with intermittent shaking every 10 minutes. After digestion, the NP cells were harvested through a 70 *μ*m cell strainer and centrifuged (1200 rpm) for 5 min. The NP cells were incubated with a complete DMEM culture medium (Gibco, USA) containing 12% heat-inactivated fetal bovine serum (FBS, Gibco, USA), streptomycin (100 mg/mL), and penicillin (100 U/mL). The NP cells were then incubated in a humidified 5% CO_2_ atmosphere. When cell growth reached 80–90% confluence, the NP cells were dispersed using 0.25% trypsin (Gibco, USA) and resuspended in the appropriate culture plates. NP cells from passages 2–5 were used for all subsequent experiments.

### 2.4. Cyclic Mechanical Stretch

**Figure 1:**
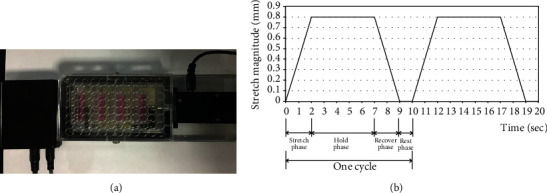
Nucleus pulposus (NP) cells loaded with cyclic mechanical stretch. (a) Computer-controlled cell stretching equipment (Mechano Culture FX2, Cell Scale, Canada). (b) Mechanical loading curve of NP cells, 10% deformation, 0.1 Hz for 8640 cycles (one cycle consisted of 2 s of stretch, 5 s of hold, 2 s of recover, and 1 s of rest).

The cyclic mechanical stretch model of NP cells in vitro was established using a cell stretching instrument (Mechano Culture FX2, Cell Scale, Canada) that was controlled by a computer. The NP cells were seeded into a sixteen-well flexible-bottom Silicone Plate (Cell Scale, Canada) at a density of 2 × 10^5^ cells/200 *μ*L in the culture medium and incubated in a humidified 5% CO_2_ atmosphere for 12 h. The cells were then subjected to cyclic tensile stress (10% deformation) of 8640 cycles at 0.1 Hz (2 s of stretch, 5 s of hold, 2 s of recover, and 1 s of rest for 1 cycle; Figure 1). As the control group, NP cells were cultured under the same conditions without any mechanical stretch. After the stretch was completed, the cells were harvested for the subsequent experiments.

### 2.5. Cell Proliferation Assay

The proliferation of NP cells was detected through MTT assay. The cells were seeded in a 96-well plate (2.5 × 10^3^ cells per well). An MTS kit was used to detect the cell growth rate based on the manufacturer's protocol (Promega, USA). The experiment was performed three times.

### 2.6. Detection of the Cell Cycle

Cells in the tensile stress and control groups were harvested, washed twice with the ice-cold PBS, and fixed with 75% ethyl alcohol at –20°C for 1 hour. The cells were then resuspended in 400 mL PBS and incubated with RNase at 37°C for 30 minutes. The cells were then incubated with propidium iodide in the dark at 4°C for 60 minutes and analyzed using a flow cytometer (Beckman, USA). The experiment was performed three times.

### 2.7. Detection of Cell Apoptosis

NP cells were washed twice in ice-cold PBS, resuspended in 400 mL binding buffer, and incubated with Annexin V-fluorescein isothiocyanate (Solarbio, China) in the dark for 15 minutes at 4°C. The NP cells were then incubated with propidium iodide in the dark at 4°C for 5 minutes and visualized in a flow cytometer (Beckman, USA). The experiment was performed three times.

### 2.8. Gene Expression Profile Microarray and Bioinformatics Analysis

The GeneChip® PrimeView™ Human Gene Expression Array (Affymetrix, Santa Clara, CA, USA) was used to analyze the gene expression profile microarray. The experiments and bioinformatics analysis were conducted by Gene Co., Ltd. (Shanghai, China). The quality of the original chip data was evaluated, including signal strength distribution, Pearson's correlation analysis, and principal component analysis. The probe signal strength that ranged in the lowest 20% was taken as background noise, and probes with a coefficient of variation greater than 25% were filtered out. After data cleaning, the remaining data were conducted to analyze the differentially expressed genes (DEGs) and function analysis. The log_2_ | fold change | ≥1.5 and a *P* value cutoff of 0.05 were used as filters to identify the DEGs. The significance enrichment analysis of Gene Ontology (GO) annotation was analyzed by Fisher's exact test. A gene set enrichment analysis of all DEGs was conducted based on the KEGG (Kyoto Encyclopedia of Genes and Genomes) database to identify significantly enriched signaling pathways.

### 2.9. Total RNA Extraction and qRT-PCR

Total RNA was extracted using TRIzol solution (Invitrogen, California, USA) based on the manufacturer's instructions. The qRT-PCR was carried out as previously described [21] using *GAPDH* (glyceraldehyde-3-phosphate dehydrogenase) as an internal control. The primers are listed in Table 1. The Ct (threshold cycle) value was analyzed based on the threshold cycles via SDS 2.3 software. Relative expression levels of target genes were normalized to the geometric mean of *GAPDH* and analyzed via the comparative threshold cycle (2^–*Δ*CT^) method.

### 2.10. siRNA (Small Interfering RNA) Transfection

siRNAs targeting *ITGA2* (siRNA-ITGA2) and *AKT* (siRNA-AKT) (GenePharma Company, Shanghai, China) were used to knock down the expression of ITGA2 and AKT proteins in NP cells. Briefly, NP cells were seeded in a 6-well plate (1 × 10^6^ cells per well). When the NP cell growth reached about 50% confluence, the cells were transfected with a 500 *μ*L mixed siRNA transfecting reagent at 37°C for 48 hours. The siRNA transfecting reagent was prepared according to the manufacturer's protocol. Briefly, 200 pmol siRNA-ITGA2 and 200 pmol siRNA-AKT were diluted with 250 *μ*L Opti-MEM I (Invitrogen, California, USA) dilution and placed at room temperature for 5 min and then mixed with 5 *μ*L Lipofectamine RNAiMAX (Invitrogen, California, USA) diluted with 250 *μ*L Opti-MEM I. The siRNA-NC was used as a negative control. The ITGA2 and AKT levels were measured 48 hours after transfection. The siRNA sequences used in this study were as follows: GAACGGGACUUUCGCAUCA for siRNA-ITGA2, ACAAGGACGGGCACATTAA for siRNA-AKT, and UUCUCCGAACGUGUCACGUTT for siRNA-NC.

### 2.11. Protein Extraction and Western Blot

NP cells were lysed using RIPA (Beyotime, Shanghai, China). The lysates were harvested through centrifugation (12,000 rpm) at 4°C for 30 min. Western blot was performed to determine the protein expression of target genes as previously described [21]. Densitometry was used to determine the band intensity using Quantity One software (Bio-Rad Laboratories, Inc., Hercules, USA). The protein expression levels were normalized against that of *β*-actin. The following primary and secondary antibodies were used: a mouse monoclonal antibody against ITGA2 (1 : 1000 dilution; Thermo Fisher Scientific, USA), a rabbit polyclonal antibody against AKT (1 : 1000 dilution; Cell Signaling Technology, Boston, MA, USA), a rabbit polyclonal antibody against phosphorylated AKT (p-AKT) (1 : 500 dilution; Abcam, St. Louis, MI, USA), a rabbit monoclonal antibody against COL2A1 (1 : 2000 dilution; Abcam, St. Louis, MI, USA), a mouse monoclonal antibody against *β*-actin (1 : 1000 dilution; Santa Cruz Biotechnology, China), a horseradish peroxidase- (HRP-) conjugated rabbit anti-mouse secondary antibody (1 : 2000 dilution; Santa Cruz Biotechnology, China), and a HRP-conjugated goat anti-rabbit secondary antibody (1 : 2000 dilution; Epitomics, Burlingame, USA).

### 2.12. Statistical Analysis

Statistical analyses were conducted through SPSS 22.0 software (IBM, Armonk, USA), and a *P* value < 0.05 was considered statistically significant. Student's *t*-test was used for the comparison of continuous variables between groups. All data were shown as the mean ± standard deviation (SD).

## 3. Results and Discussion

### 3.1. Morphological Observation of NP Cells Responding to the Cyclic Mechanical Stimuli

Biomechanical factors have been reported to be remarkably associated with NP degeneration [8, 9]. Biomechanics influence the multiple biological functions of NP cells, such as regulating the balance of ECM anabolism and catabolism, the abnormal expression of growth factors and inflammatory molecules, and the nutrition and metabolism of NP cells [8, 9]. As an important component of IVDs, the NP region is subjected to a different tension, pressure, and shear force under different physiological states. These different biomechanics lead to different effects on the function and degeneration of NP cells [7].

**Figure 2:**
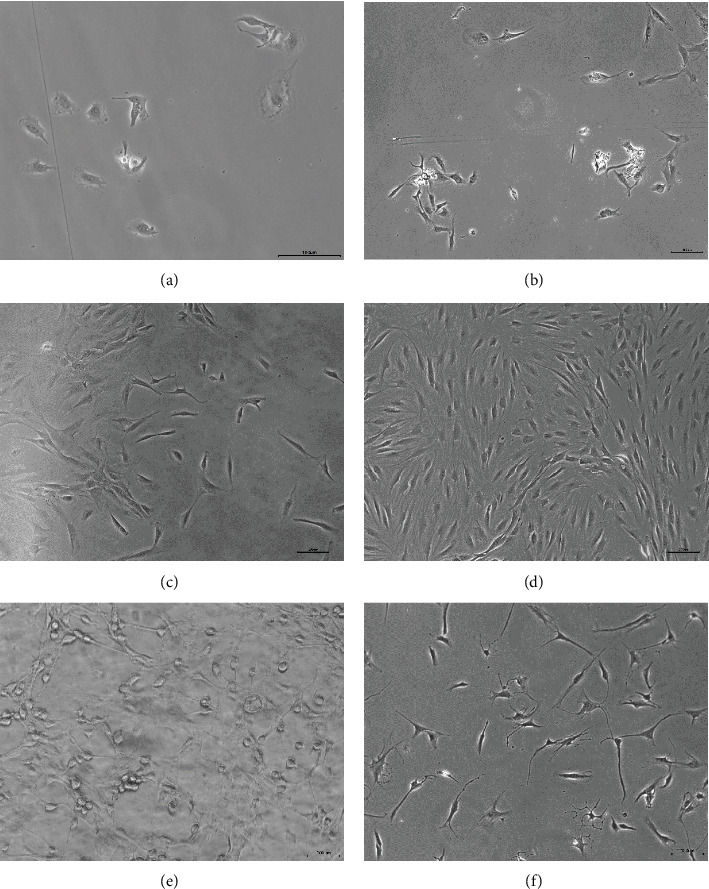
NP cells observed under an inverted phase contrast microscope. (a) NP cells cultured on day 5. (b) NP cells cultured on day 9. (c) NP cells cultured on day 15. (d) NP cells cultured on day 25. (e) Passage 3 NP cells loaded with cyclic mechanical stretch. (f) Passage 3 NP cells of the control group.

This study investigated the effect of cyclic mechanical stretch on the biological function of NP cells. As observed under a microscope, NP cells were spindle-shaped or irregular in shape and appeared singly or as 2- to 4-cell colonies scattered at the bottom of the bottle on day 5 of culture (Figure 2(a)). On day 9 of culture, the number of NP cells increased gradually, and the cells from each colony were closely connected (Figure 2(b)). On day 15 of culture, the culture reached 40–60% confluence with good morphology and refraction (Figure 2(c)). On culture day 25, cell growth reached 80–90% confluence (Figure 2(d)). NP cells from passages 2–5 were used to load the cyclic mechanical stretch. Results showed that NP cells in the tensile stress group were irregularly shaped with a full growth state and good refraction (Figure 2(e)), whereas the condition of NP cells in the control group was not good, with longer secondary processes and appeared aging (Figure 2(f)).

### 3.2. Cyclic Mechanical Stretch Promotes NP Cell Proliferation and Cell Cycle Progression

**Figure 3:**
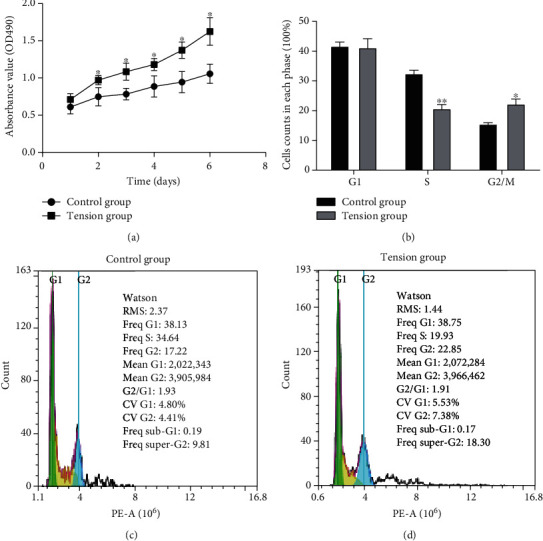
Effects of cyclic mechanical stretch on the proliferation and cell cycle of NP cells. (a) The MTS assay showing that cyclic mechanical stretch promoted NP cell proliferation. (b) Cyclic mechanical stretch significantly promoted the progression of NP cells from the S phase to the G2/M phase. (c) Flow cytometric results showing the percentages of NP cells in the G1, S, and G2/M phases in the control group. (d) Flow cytometric results showing the percentages of NP cells in the G1, S, and G2/M phases in the tensile stress group. ^∗^*P* < 0.05. ^∗∗^*P* < 0.01.

The effect of cyclic mechanical stretch on the proliferation of NP cells was then evaluated. As shown in Figure 3(a), NP cells exhibited a significantly higher growth rate in the tensile stress group compared to the control group (*P* < 0.05). We then analyzed the effects of cyclic mechanical stretch on the cell cycle by flow cytometry. The percentages of NP cells in the G1, S, and G2/M phases in the tensile stress and control groups were 41.22 ± 5.17% vs. 41.71 ± 2.45%, 20.74 ± 2.32% vs. 32.47 ± 1.95%, and 22.27 ± 2.91% vs. 15.53 ± 0.78%, respectively (Figures 3(c) and 3(d)). The percentage of NP cells in the S phase was remarkably lower in the tensile stress group compared to the control group (*P* < 0.01, Figure 3(b)). The percentage of NP cells in the G2/M phase was significantly higher in the tensile stress group than in the control group (*P* < 0.05, Figure 3(b)). The results revealed that cyclic mechanical stretch significantly promoted the cell cycle transition of NP cells from the S phase to the G2/M phase.

### 3.3. Cyclic Mechanical Stretch Inhibits NP Cell Apoptosis

**Figure 4:**
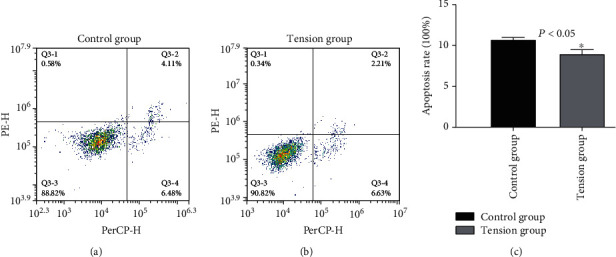
Effects of cyclic mechanical stretch on apoptosis of NP cells. (a) Percentages of apoptotic NP cells in the control group analyzed by flow cytometry. (b) Percentages of apoptotic NP cells in the tensile stress group analyzed by flow cytometry. (c) Cyclic mechanical stretch suppressed apoptosis of NP cells. ^∗^*P* < 0.05.

We analyzed the effects of cyclic mechanical stretch on the NP cell apoptosis. The apoptosis percentages of NP cells in the tension and control groups were 8.99 ± 0.88% and 10.75 ± 0.40%, respectively (Figures 4(a) and 4(b)). A lower percentage of apoptotic NP cells was found in the tensile stress group compared with the control group (*P* < 0.05, Figure 4(c)), indicating that cyclic mechanical stretch inhibited apoptosis in NP cells.

These results suggested that cyclic mechanical stretch may affect the degeneration of the NP by regulating the proliferation, cell cycle, and apoptosis of NP cells. Matsumoto et al. reported that cyclic mechanical stretch increases the growth rate of NP cells in vitro [22]. On the contrary, Yang et al. and Wang et al. reported that abnormal mechanical stretch stress led to excessive apoptosis and degeneration of NP cells [23, 24]. Cyclic mechanical tension with 20% elongation induced NP cell apoptosis [25]. When comparing the mechanical loading parameters of high amplitude (>10%), low frequency (<0.1 Hz), and long duration (>24 hours) in the previous study, the tensile stress used in the current study was applied at 0.1 Hz frequency for 24 h to produce 10% deformation. Together with these reported studies, our findings indicate that it is possible that high mechanical stretch would promote NP apoptosis and degeneration, while moderate mechanical stretch would inhibit NP cell apoptosis, promote NP cell proliferation, and potentially ameliorate NP degeneration.

### 3.4. Differentially Expressed Genes in Cyclic Mechanical Stretch-Stimulated NP Cells

**Figure 5:**
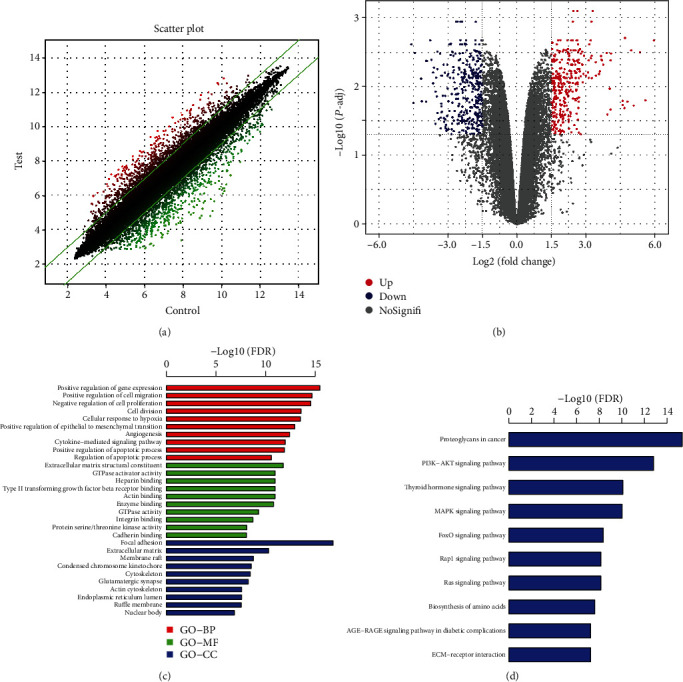
Differential mRNA expression between the tensile stress and control groups. (a) Scatter plot showing the differential expression of mRNAs between the tensile stress and control groups. Red and green plots represent up- and downregulated genes, respectively. (b) The volcano plot showing mRNAs with differential expression between the two groups. Red and blue plots represent up- and downregulated genes, respectively. (c) Significance enrichment analysis of Gene Ontology (GO) annotation results. (d) A gene set enrichment analysis of all the differentially expressed genes showing the significantly enriched pathways based on the KEGG database. BP: biological process; MF: molecular function; CC: cellular component.

To shed light on the mechanism by which cyclic mechanical stretch affects NP cells, microarray analyses were performed to examine the effector genes that respond to cyclic mechanical stretch. Genes that showed log_2_ | fold change | ≥1.5 upregulation or downregulation in all the six paired samples were defined as DEGs or effector genes. The results of the microarray analyses revealed a total of 689 candidate genes that showed significant differential expression between the tension group and the control group (*P* < 0.05), of which 333 genes were upregulated and 356 genes were downregulated (Figures 5(a) and 5(b)). The top ten upregulated genes were *SPP1* (secreted phosphoprotein 1), *RANBP3L* (RAN-binding protein 3-like), *SLC6A12* (solute carrier family 6 member 12), ITGA2 (integrin alpha 2), *C2CD4B* (C2 calcium-dependent domain containing 4B), *RGCC* (regulator of cell cycle), *SLCO4A1* (solute carrier organic anion transporter family member 4A1), *SLC16A6* (solute carrier family 16 member 6), *PGM2L1* (phosphoglucomutase 2-like 1), and *NTN1* (netrin 1). The top ten downregulated genes were *MEST* (mesoderm specific transcript), *KIAA0101*, *GRB14* (growth factor receptor bound protein 14), *LGR5* (leucine-rich repeat-containing G protein-coupled receptor 5), *CPA4* (carboxypeptidase A4), *TAGLN* (transgelin), *TOP2A* (topoisomerase DNA II alpha), *CD24* (CD24 molecule), *RRM2* (ribonucleotide reductase M2), and *RCAN2* (regulator of calcineurin 2).

We then performed GO annotation analysis, which consists of three main branches, namely, biological process (BP), molecular function (MF), and cellular component (CC).  Figure 5(c) presents the top ten significantly enriched BP, MF, and CC associated with cyclic mechanical stretch stimulation. GO annotation analysis showed that the main genes from the BP category were positive regulation of gene expression, cell proliferation and division, and apoptotic processes; the genes from the MF category were mainly ECM structural constituents; and those from the CC category included focal adhesion and ECM. Abnormal NP cell proliferation, apoptosis, and ECM metabolism have been shown to result in NP degeneration [8]. The microarray results revealed that the genes with significant differential expression that were involved in these biological functions and processes are closely related to NP degeneration.

In addition, we analyzed the involvement of all DEGs associated with signaling pathways based on the KEGG database.  Figure 5(d) shows the top ten enriched signaling pathways associated with cyclic mechanical stretch stimulation, and these signaling pathways included proteoglycans in cancer, PI3K/AKT, thyroid hormone, MAPK, FoxO, Rap1, Ras, biosynthesis of amino acids, AGE-RAGE signaling pathway in diabetic complications, and ECM-receptor interaction. Of these, the PI3K/AKT pathway has previously been reported to regulate cellular functions such as cell proliferation, growth, cell cycle, and apoptosis [15, 16]. The PI3K/AKT pathway was reported to participate in the regulation of NP cell apoptosis, senescence, and ECM degradation and is significantly associated with NP degeneration [19, 26, 27]. Based on the above experimental results, it is reasonable that the PI3K/AKT signaling process might play important roles in the cyclic mechanical stretch-mediated effects on NP cells.

A total of 31 genes involved in the PI3K/AKT signaling pathway were uncovered to show significant differential expression between the two groups. Of these, 18 genes, including *ITGA2*, *ITGA10* (integrin subunit alpha 10), *BCL2L11* (BCL2-like 11), *COL2A1* (collagen type II alpha 1), and *FGF1* (fibroblast growth factor 1), which are positive effectors in this pathway, were markedly upregulated. And conversely, 13 genes that negatively regulate the pathway, such as *MYC* (MYC proto-oncogene), *IL7R* (interleukin 7 receptor), and *THBS1* (thrombospondin 1), were markedly downregulated. We further conducted qRT-PCR to confirm the microarray results. Results showed that the mRNA levels of *ITGA2*, *FGF1*, *COL2A1*, and *BCL2L11* were significantly increased, and the *MYC* and *IL7R* mRNA levels were markedly decreased in NP cells in the tension group compared with the control group (log_2_ | fold change | ≥1.5, *P* < 0.05, Table 2). These results further supported our findings from the microarray data.

### 3.5. Involvement of the ITGA2/PI3K/AKT Pathway in the Cyclic Mechanical Stretch-Mediated Effects on NP Cells

Integrins are widely considered the predominant mechanoreceptors during the process of mechanical transduction in IVD cells [28]. Mechanosensing in NP cell loading with dynamic mechanics occurs via integrin signaling [28]. The integrin pathways play an important role in the susceptibility of notochordal cells to mechanical loading-induced IVDD [29]. ITGA2, also known as integrin *α*2, encodes the alpha subunit of a transmembrane receptor for collagens and other related proteins. ITGA2 forms a heterodimer with an integrin beta subunit, mediating the adhesion of multiple cell types to the ECM. Integrin *α*2*β*1 participates in the estrogen-intervened PI3K/AKT pathway regulating the antiapoptotic effect of intervertebral disc aging and degeneration and participates in FAK/PI3K signaling regulating adherence capacity and anoikis of NP cells in IVD degeneration [30]. The FAK/PI3K/AKT pathway has been reported to acts as integrin-dependent signaling in regulating apoptosis of NP cells during the process of disc degeneration [31]. Integrins function synergistically with SYND4, and FAK/PI3K signaling participates in regulating adherence capacity and anoikis of NP cells in IVD degeneration [32]. Yan et al. reported that static compression loading upregulated integrin *α*2*β*1 expression and downregulated *α*10*β*1 expression in NP cells [33]. Compression loading altered the expression of collagen, MMP, and integrin and promoted integrin *α*2*β*1 signaling in IVD [33]. However, the role of ITGA2 in the effect of cyclic mechanical stretch on NP cell degeneration has not been elucidated.

**Figure 6:**
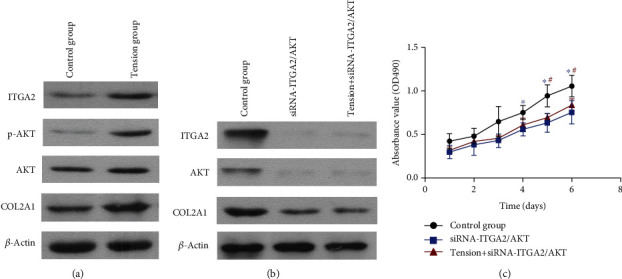
Involvement of the PI3K-AKT pathway in the cyclic mechanical stretch-mediated effects on NP cells. (a) Cyclic mechanical stretch promoted the PI3K-AKT pathway in NP cells. The protein expression of ITGA2, p-AKT, AKT, and COL2A1 was upregulated in the tensile stress group. (b) Inhibition of the PI3K-AKT pathway. Western blot analyses showing downregulation of ITGA2 and AKT protein expression in NP cells transfected with siRNA-ITGA2/AKT. COL2A1 protein expression was remarkably decreased. Cyclic mechanical stretch did not significantly affect the expression of ITGA2, AKT, and COL2A1 in NP cells transfected with siRNA-ITGA2/AKT. (c) Downregulation of the PI3K-AKT pathway significantly inhibited the proliferation of NP cells. Cyclic mechanical stretch did not significantly affect the proliferation of NP cells when the PI3K-AKT pathway was inhibited.

In this study, Western blot was performed to further corroborate the effects of cyclic mechanical stretch on the ITGA2/PI3K/AKT signaling process in NP cells. The results of the Western blot revealed that the expression levels of the ITGA2 and p- (phosphorylated-) AKT proteins were remarkably higher in the tensile stress group compared to the control group (Figure 6(a)), indicating that cyclic mechanical stretch remarkably promotes the ITGA2/PI3K/AKT signaling pathway in NP cells.

To further substantiate the functional effect of cyclic mechanical stretch on the ITGA2/PI3K/AKT signaling pathway, we used siRNAs to knock down the protein expression of the key molecules in this pathway in NP cells, i.e., ITGA2 and AKT. Results of the Western blot analysis showed that ITGA2 and AKT proteins were markedly decreased (Figure 6(b)), indicating that the ITGA2/PI3K/AKT signaling pathway was inhibited. And the ITGA2 and AKT expression showed no obvious change when the NP cells were loaded with cyclic mechanical stretch. We then determined the NP cell proliferation. Inhibition of this pathway significantly inhibited the proliferation of NP cells (Figure 6(c)), whereas cyclic mechanical stretch did not significantly affect the proliferation of NP cells transfected with siRNA-ITGA2/AKT. These results indicate that the ITGA2/PI3K/AKT signaling pathway plays a key role in the effect of cyclic mechanical stretch on NP cell proliferation.

Interestingly, we found that the cyclic mechanical stretch elevated COL2A1 protein expression in NP cells (Figure 6(a)). And COL2A1 protein expression was remarkably decreased after the knockdown of the ITGA2/PI3K/AKT signaling pathway (Figure 6(b)). The ECM of NP consists of collagens and proteoglycans, of which aggrecan is one of the most abundant proteoglycans in NP tissues [34, 35]. COL2A1 is a main component of the ECM in NP tissues and has been considered a key molecule in IVD degeneration [12]. Many ECM molecules are altered in degenerated NP, and collagen type II to collagen type I is one such predominant shift in the NP [36]. These results indicated that cyclic mechanical stretch may regulate COL2A1 expression through the ITGA2/PI3K/AKT signaling pathway and ameliorate the degeneration of NP cells. Further studies will investigate the molecular mechanism of cyclic mechanical stretch in regulating the ECM and inflammation of NP cells in the process of NP degeneration.

## 4. Conclusion

In conclusion, we provided evidence that cyclic mechanical stretch promoted NP cell proliferation and cell cycle transition from the S phase to the G2 phase and inhibited apoptosis of NP cells. Results of the microarray analysis revealed that 689 genes had significant differential expression. Enrichment analysis of all the DEGs in regulatory signaling pathways found the PI3K/AKT pathway. The results further revealed that cyclic mechanical stretch promoted cell proliferation and COL2A1 expression through regulation of the ITGA2/PI3K/AKT signaling pathway and ameliorates the degeneration of NP cells. These results of our study may provide potential targets and the possibility of treating IVDD disease by reversing degenerative changes.

## Figures and Tables

**Table 1:** Primers used for qRT-PCR analysis.

Gene	Primer	Lengths of products
ITGA2	Forward 5′-CAAACCTCTGCAAACCCAGC-3′ Reverse 5′-TCGGTTCTCAGGAAAGCCAC-3′	277 bp
FGF1	Forward 5′-TTACCACGCCTTGACCTTCC-3′ Reverse 5′-ATGGTATCCCCTCAGCCAGT-3′	186 bp
COL2A1	Forward 5′-ATGAGGGCGCGGTAGAGA-3′ Reverse 5′-GCCAGCCTCCTGGACATC-3′	190 bp
BCL2L11	Forward 5′-GTATTCGGTTCGCTGCGTTC-3′ Reverse 5′-GGAAGCTTGTGGCTCTGTCT-3′	244 bp
MYC	Forward 5′-ACTAACATCCCACGCTCTGA-3′ Reverse 5′-AAACCGCATCCTTGTCCTGT-3′	220 bp
IL7R	Forward 5′-TCCAACCGGCAGCAATGTAT-3′ Reverse 5′-CACACAGGCCAAGATGACCA-3′	203 bp
GAPDH	Forward 5′-AATGGGCAGCCGTTAGGAAA-3′ Reverse 5′-GCCCAATACGACCAAATCAGAG-3′	166 bp

**Table 2:** Validation of mRNA expression genes in the PI3K-AKT signaling pathway using qRT-PCR on the same sample set of the microarray study.

Gene	Fold change^a^ (microarray)	Fold change (qRT-PCR)	Fold change (qRT-PCR replication)
ITGA2	17.05	30.12	26.59
FGF1	2.69	3.24	5.36
COL2A1	2.15	2.97	3.51
BCL2L11	3.04	2.31	2.83
MYC	-3.35	-3.59	-3.81
IL7R	-3.29	-4.29	-2.73

^a^Fold change represents the difference multiple of gene expression between the tension group and the control group.

## Data Availability

The data used to support the findings of this study are included within the article.
